# Efficacy and safety of ultrasound-assisted wound debridement in the treatment of diabetic foot ulcers: a systematic review and meta-analysis of 11 randomized controlled trials

**DOI:** 10.3389/fendo.2024.1393251

**Published:** 2024-05-01

**Authors:** Erhao Liu, Xiaojie Hu, Wenke Zhang, Wen Xiao, Yiting Shen, Yu Luo, Zeyu Zheng, Pengfei Zhou, Youcheng He, Huafa Que

**Affiliations:** ^1^ Department of Traditional Chinese Surgery, Longhua Hospital Shanghai University of Traditional Chinese Medicine, Shanghai, China; ^2^ Longhua Medical College, Shanghai University of Traditional Chinese Medicine, Shanghai, China

**Keywords:** ultrasound-assisted wound debridement, diabetic foot ulcers, systematic review, meta-analysis, randomized controlled trial

## Abstract

**Objective:**

Research data suggests that ultrasound-assisted wound debridement (UAWD) can effectively promote the healing of diabetic foot ulcers (DFU). However, existing research is not consistent with this viewpoint. Therefore, we conducted this study to investigate the effect of UAWD on the healing of diabetic foot ulcers.

**Methods:**

From the establishment of the database to January 2024, we searched 8 databases to study the effectiveness and safety of UAWD in the treatment of DFU. Two authors independently screened the qualifications of the articles, while two authors extracted relevant data. Statistical analysis was conducted using Review Manager 5.4 and STATA 18.0 software.

**Results:**

A total of 11 randomized controlled studies were included, with 6 countries and 696 participants participating. Our findings showed that UAWD was associated with a significant benefit in healing rate (OR = 2.60, 95% CI: [1.67, 4.03], P < 0.0001, I^2 =^ 25%), wound healing time (MD = -11.94, 95% CI: [-23.65, -0.23], P = 0.05, I^2 =^ 99%), percentage reduction in wound size (MD = 14.2, 95% CI: [10.8, 17.6], P = 0.47, I^2 =^ 32%), effectiveness of treatment (OR = 10.3, 95% CI: [4.68, 22.66], P < 0.00001, I^2 =^ 0%). Moreover, UAWD did not cause any significant adverse reactions. However, there was no obvious difference in wound blood perfusion (MD = 0.25, 95% CI: [-0.01, 0.52], P = 0.06, I^2 =^ 90%), transcutaneous oxygen partial pressure (MD = 14.34, 95% CI: [-10.03, 38.71], P = 0.25, I^2 =^ 98%).

**Conclusion:**

UAWD can significantly improve wound healing rate, shorten wound healing time, accelerate wound area reduction, and improve clinical treatment effectiveness without significant adverse reactions. Although there is no significant difference in transcutaneous oxygen pressure and wound blood flow perfusion between UAWD and SWC. So we look forward to more scientifically blinded, placebo-controlled, high-quality studies in the future, to enable researchers to obtain more complete and accurate analytical data, in order to improve the scientific and credibility of the evidence.

**Systematic review registration:**

https://www.crd.york.ac.uk/prospero/, identifier CRD42024501198.

## Introduction

Diabetic foot ulcers (DFU) refer to diabetic patients that present with tissue loss below the ankle that fails to heal, and this can be due to distal peripheral arteriopathy and peripheral neuropathy of the lower extremities caused by tissue ischemia, sensory abnormalities, and so on, which and can lead to a series of foot infections, ulcers, necrosis and other diseases with tissue, and in severe cases, can lead to disability and death (1). Surveys have shown that between 19% and 34% of diabetes patients will develop DFU during their lifetime (2). With the continuous improvement of social living standards and medical security, the extension of human life expectancy and the gradual aging of the population, the number of patients with diabetes mellitus and DFU will increase day by day in the future, and the costly treatment will bring huge economic losses to the patients and a heavy burden on the country’s medical resources (3).

Current basic clinical treatments for DFU include improving glycemic control, managing infection, metabolic regulation, offloading of wounds, dressing therapies, the use of vasodilating and antiplatelet drugs etc. And advanced therapies include UAWD (4), negative pressure wound therapy (5), hyperbaric oxygen therapy, novel wound dressing, autologous platelet rich plasma, and stem cell therapy, etc (6). In recent years, many studies using UAWD for the treatment of DFU have been increasing and have achieved significant clinical efficacy, but existing research is not consistent with this viewpoint.

Based on these previous findings, we conducted this systematic review and meta-analysis to clarify the efficacy and safety of UAWD in the treatment of DFU.

## Methods

### Protocol and registration

This study has followed the Preferred Reporting Items for Systematic Reviews and Meta-Analysis (PRISMA) guidelines (7), and the study was registered on the PROSPERO platform (CRD42024501198).

### Search strategy

The PubMed, Embase, Web of Science, Cochrane Library, China National Knowledge Internet (CNKI), Wan-Fang digital database, VIP Database for Chinese Technical Periodicals (CQVIP), and China Biology Medicine disc (CBM) were searched for Chinese or English studies from inception to January 2024. The following search terms were used: “diabetic foot” or “foot, diabetic” or “diabetic feet” or “feet, diabetic” or “foot ulcer, diabetic”, and “ultrasound-assisted wound debridement” or “ultrasonic debridement” or “ultrasound debridement”. The detailed search strategies are described in **Supplementary Material 1**. To prevent omissions in the literature, we searched for references to systematic reviews and meta-analysis articles in related fields. All retrieved literatures were imported into the Endnote X9 software (Thomson ResearchSoft, Stanford, CA, United States).

### Selection and eligibility criteria

Two authors (Yu Luo, Zeyu Zheng) independently screened for suitability from the original collected literatures, they would discuss with each other if there were differences, and a third author (Huafa Que) was consulted if differences could not be resolved. This meta-analysis included published studies that met the following selection criteria: (i) the subjects must be patients with DFU; (ii) patients in experimental group underwent UAWD and those in the control group were treated with placebo or standard wound care (SWC). SWC included wound irrigation with normal saline, sharp debridement, autolytic debridement, obtaining appropriate tissue/bone culture, and so on. Exclusion criteria were the following: (i) review articles, mate-analysis, conference papers, retrospective studies, cross-sectional studies, cross-RCTs, etc; (ii) studies lacking sufficient data; (iii) *in vitro* or animal experiments; (iv) studies on patients with gestational diabetes; (v) duplicate studies.

### Date extraction and quality assessment

Two authors (Wenke Zhang, Pengfei Zhou) independently extracted the following data from each study: first author, publication year, country, study type, sample size, treatment details (UAWD, SWC or placebo), observation time, primary outcomes (healing rate, wound healing time, percentage reduction in wound size, effectiveness of treatment (the percentage of patients with effective treatment to the total number of patients), wound blood perfusion, transcutaneous oxygen partial pressure). Any differences and disagreements in the extracted data were resolved by a third author (Huafa Que).

The Cochrane Risk Bias Tool version 2.0 (8) was used to assess the quality of the included studies, two researchers (Wen Xiao, Yiting Shen) independently assessed them from seven aspects: random sequence generation, allocation concealment, blinding of participants and personnel, blinding of outcome assessment, incomplete outcome data, selective reporting, and other bias.

### Statistical analysis

One author (Xiaojie Hu) used Review Manager (version 5.4, the Nordic Cochrane Centre, Copenhagen, Denmark) and Stata (version 18.0, the StataCorp LP, USA) to analyze data extracted from included literatures. For continuous variables, mean difference (MD) with 95% confidence intervals (95% CI) were reported as the effect size. Meanwhile, the dichotomous data were selected odds ratio (OR) as the effect size. Moreover, the statistical heterogeneity was tested by using the Cochran Q-test and I^2^. When I^2^ < 50%, we considered the heterogeneous was acceptable and a fixed effect model was used. Otherwise, the random effect was taken into consideration (I^2^ ≥ 50%). Subgroup analysis was performed for some of the results. Sensitivity analysis is used to evaluate the robustness of results. In addition, Begg’s test and Egger’s test were used to assess the possibility of publication bias. The significant difference level was set at P < 0.05.

## Results

### Identification and selection of studies

**Figure 1:**
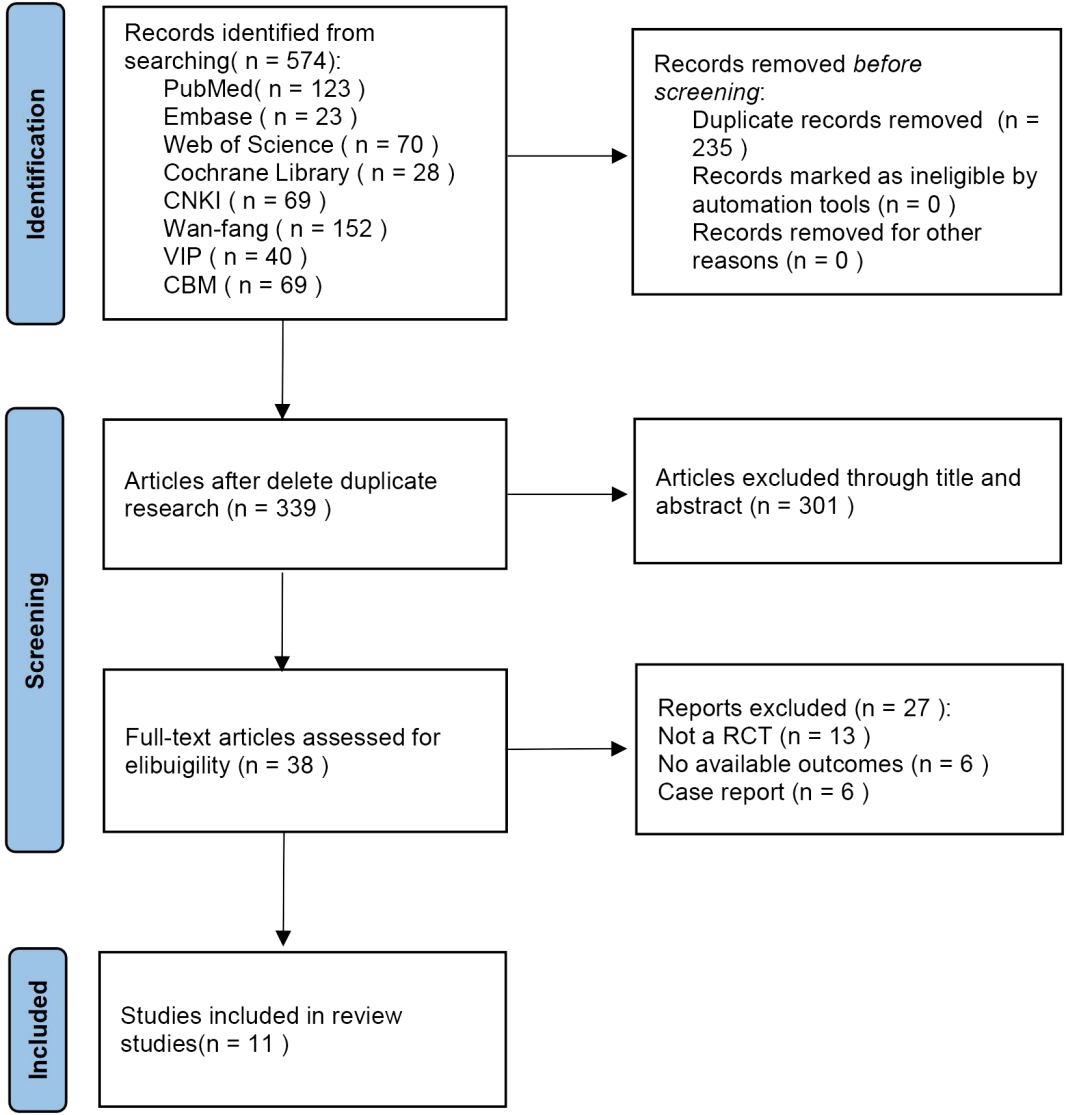
Flow diagram of systemic review procedure.

The initial literature searches retrieved 574 records from the 8 databases. After we excluded 235 duplicates, 339 literatures remained. By reading the titles and abstracts of the literatures, 301 literatures were excluded that did not meet the inclusion criteria. Afterward, among the remaining 38 studies, 27 studies were excluded by reading full text. Eventually, 11 eligible studies were included in the meta-analysis (**Figure 1**).

### Characteristics and quality assessment of studies

**Figure 2:**
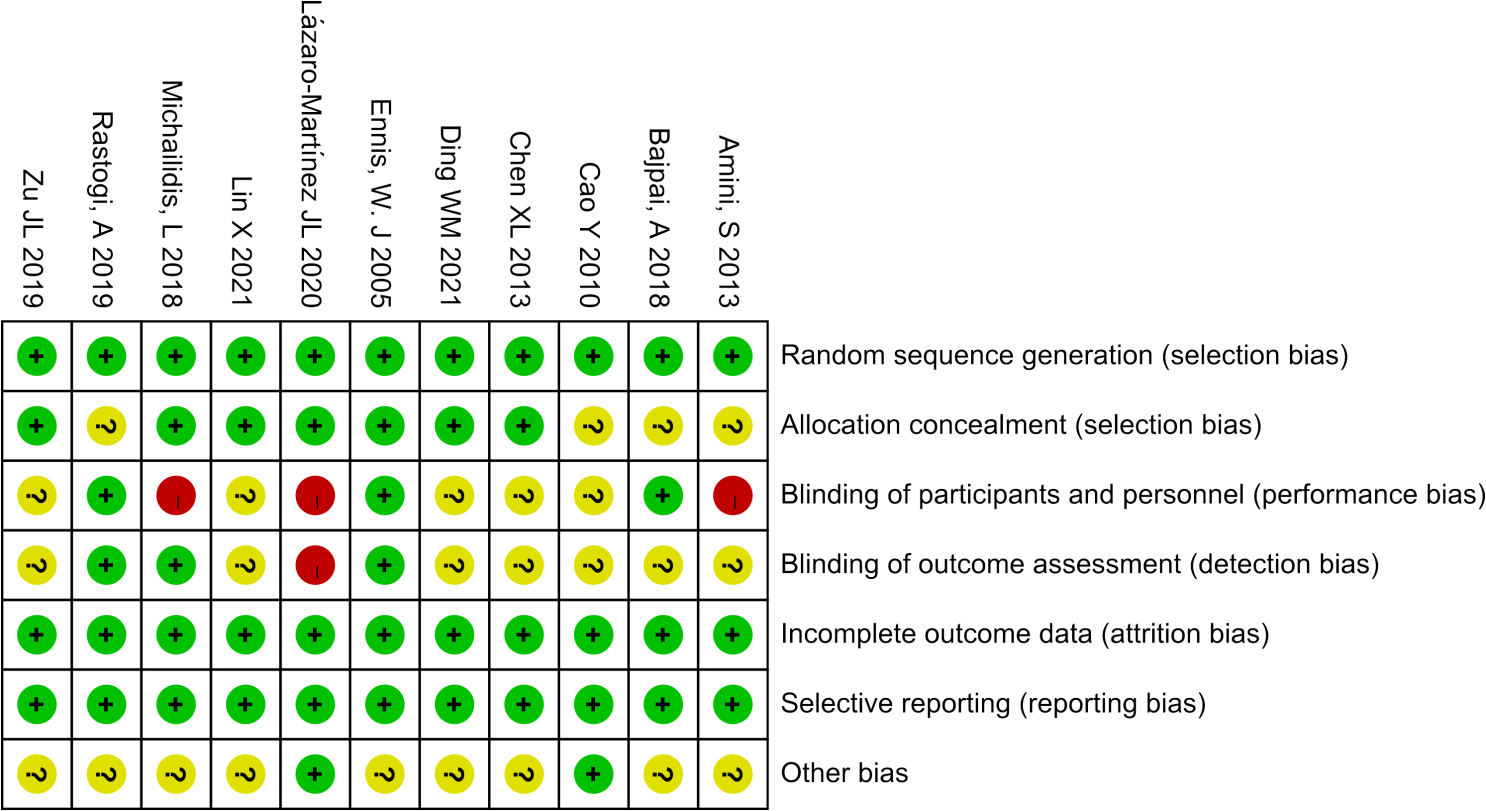
Risk of bias summary.

**Figure 3:**
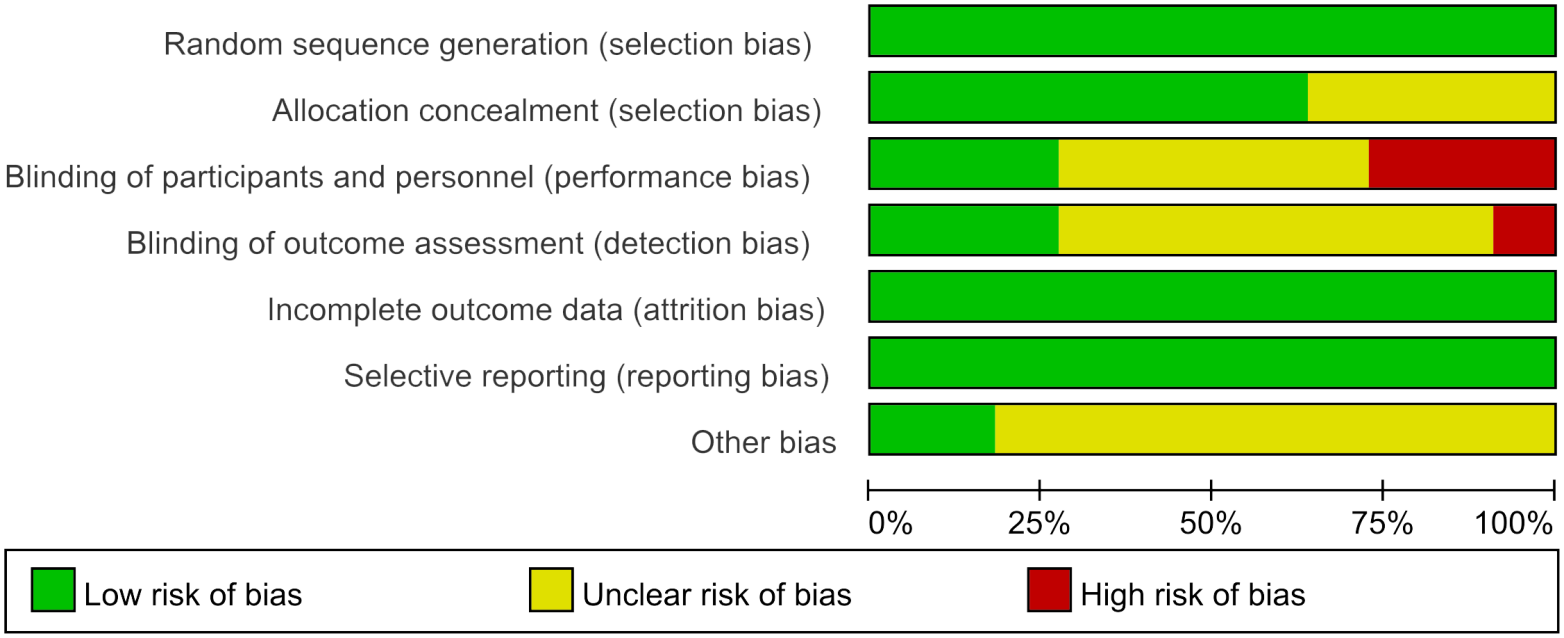
Risk of bias graph.

We eventually included 11 (9–19) studies that encompassed 696 patients, the characteristics of the studies were showed in **Table 1**. Three studies (10, 12, 14) compared UAWD with placebo, there were two studies (10, 12) were designed double-blind RCTs and two studies (11, 14) were designed single-blind RCTs among the 11 studies. These studies were conducted in Spain, India, Australia, USA, Iran, China. One of the studies (11) had 10 patients, but these patients had 14 wounds, with a total of 8 wounds in the patients in the UAWD group and 6 in the SWC group. All studies had an observation period of between 2 weeks and 6 months. All of 11 RCTs were assessed by using The Cochrane Risk Bias Tool, the details of assessing risk of bias are demonstrated in **Figures 2** and **3**.

**Table 1:** Characteristics of the included studies.

Study(author/year)	Country	Study design	Total population(n)	Experimental group,n	Control group,n	Outcomes	Observation time
Lázaro-Martínez JL 2020 (9)	Spain	RCT	51	UAWD,27	SWC.24	①②	6 weeks
Rastogi, A 2019 (10)	India	RCT	60	UAWD,34	Placebo,26	①③	4 weeks
Michailidis, L 2018 (11)	Australia	RCT	10(14 wounds)	UAWD,8	SWC,6	①②	6 months
Ennis, W. J 2005 (12)	USA	RCT	55	UAWD,27	Placebo,28	①	12 weeks
Amini, S 2013 (13)	Iran	RCT	40	UAWD,20	SWC,20	①③	6 months
Bajpai, A 2018 (14)	USA	RCT	8	UAWD,4	Placebo,4	①	12 weeks
Ding WM 2021 (15)	China	RCT	68	UAWD,34	SWC,34	②⑤	2 weeks
Cao Y 2010 (16)	China	RCT	24	UAWD,12	SWC,12	③⑤⑥	20 days
Chen XL 2013 (17)	China	RCT	62	UAWD,29	SWC,33	②③⑥	6 weeks
Lin X 2021 (18)	China	RCT	78	UAWD,39	SWC,39	①②④	NA
Zu JL 2019 (19)	China	RCT	240	UAWD,120	SWC,120	①②④	3 month

①healing rate; ②Wound healing time; ③Percentage reduction in wound size; ④Effectiveness of treatment;⑤Wound blood perfusion; ⑥Transcutaneous oxygen partial pressure.

### Healing rate

**Figure 4:**
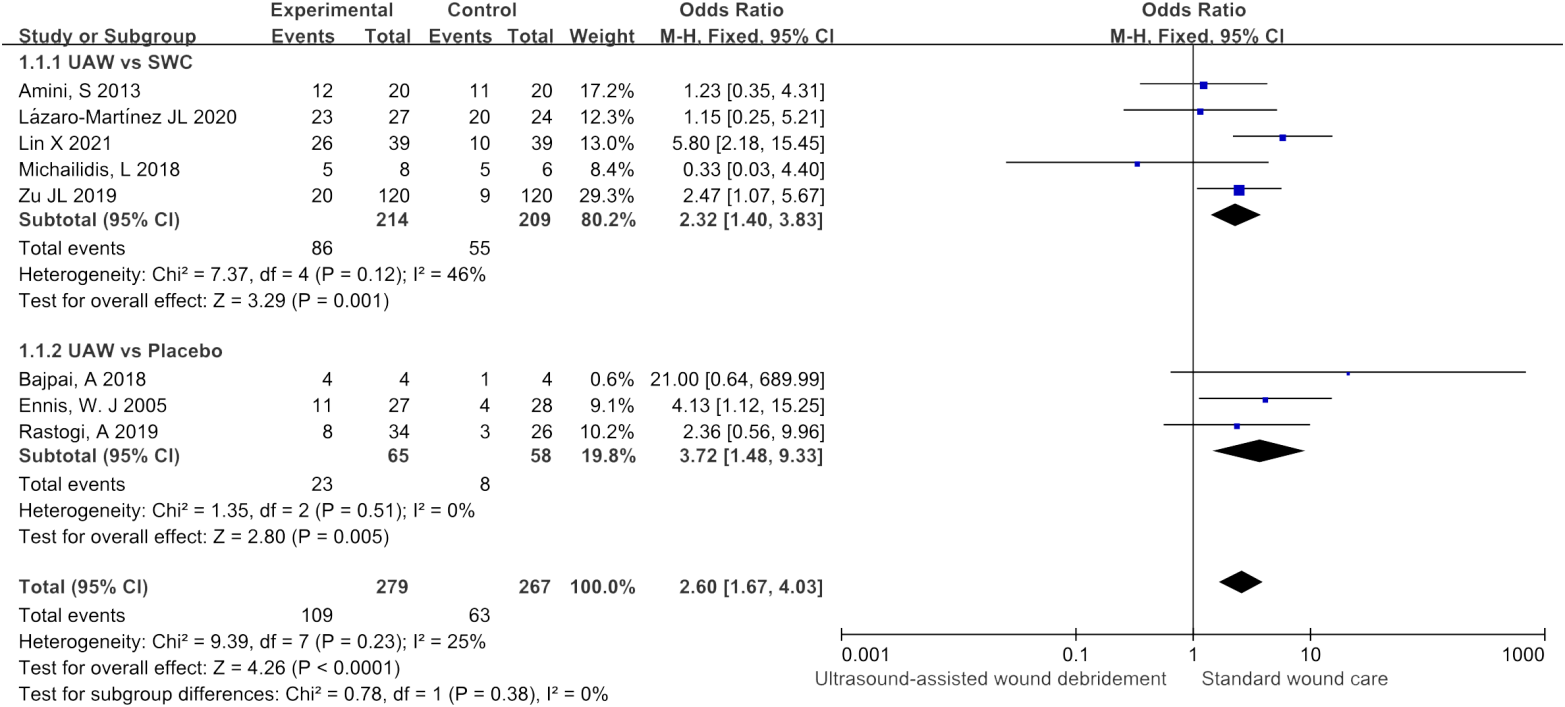
Forest plots of healing rate.

There were eight studies (9–14, 18, 19) that included wound healing rate as an observational indicator, five (9, 11, 13, 18, 19) of which were comparisons between UAWD and SWC, and three (10, 12, 14) of which were comparisons between UAWD and placebo. The overall results of the meta-analysis showed that UAWD was associated with a significant benefit in terms of the proportion of patients who had wound healing (OR = 2.60, 95% CI: [1.67, 4.03], P < 0.0001, I^2 =^ 25%). Subgroup analysis showed a higher rate of wound healing for UAWD versus SWC (OR = 2.32, 95% CI: [1.40, 3.83], P = 0.001, I^2 =^ 46%) and placebo (OR = 3.72, 95% CI: [1.48, 9.33], P = 0.005, I^2 =^ 0%) (**Figure 4**).

### Wound healing time

**Figure 5:**
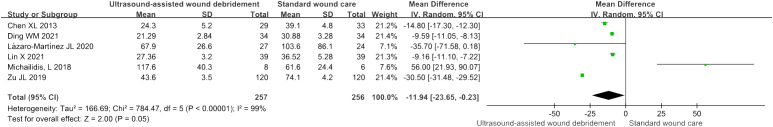
Forest plots of wound healing time.

Six studies (9, 11, 15, 17–19) investigated the effects of wound healing time by comparing UAWD with SWC, meta-analysis results showed significant difference in two therapies (MD = -11.94, 95% CI: [-23.65, -0.23], P = 0.05, I^2 =^ 99%) (**Figure 5**). The results of the sensitivity analysis showed that excluding any of the studies had no obvious effect on the MD values. The detailed data is described in **Supplementary Material 2**.

### Percentage reduction in wound size

**Figure 6:**
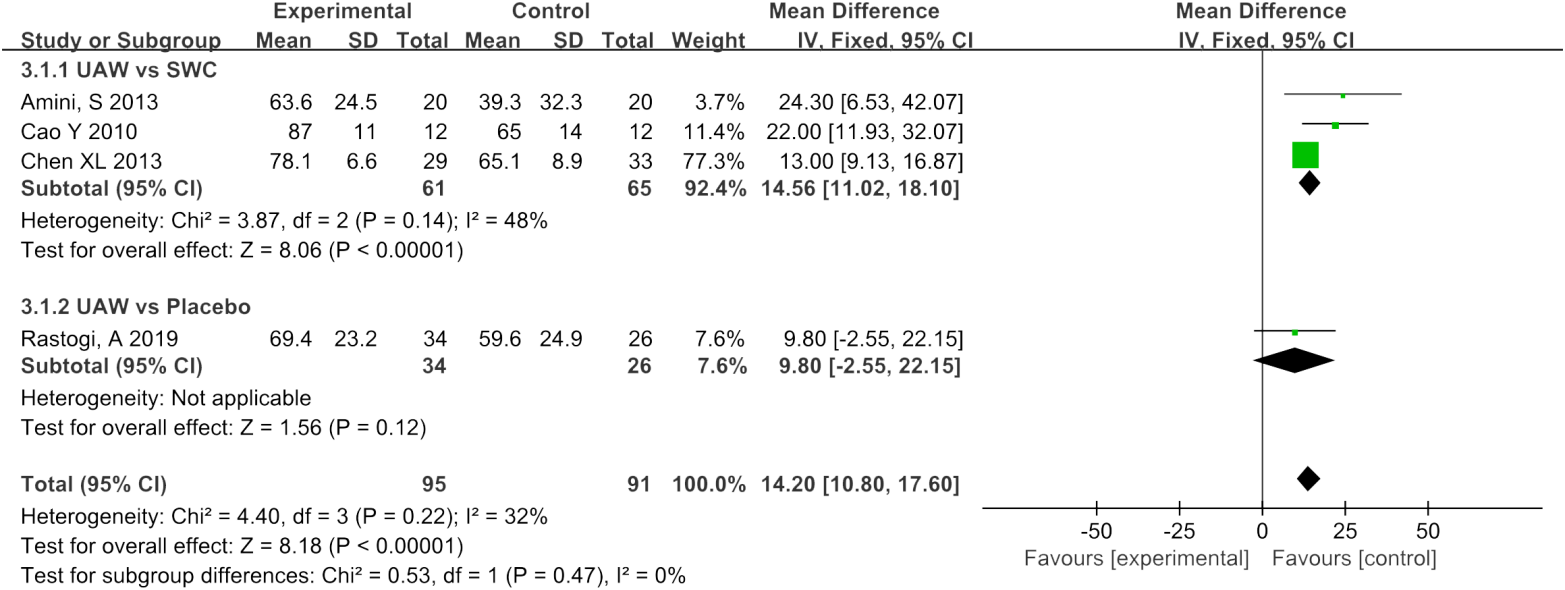
Forest plots of percentage reduction in wound size.

Four RCTs (10, 13, 16, 17) used percentage reduction in wound size as an observable indicator of efficacy. The meta-analysis of this data estimated the pooled MD at 14.2, 95% CI: [10.8, 17.6], P < 0.00001, I^2 =^ 32%. Results of subgroup analysis indicated that UAWD showed a significant improvement in percentage reduction in wound size when compared to SWC (MD = 14.56, 95% CI: [11.02, 18.1], P < 0.00001, I^2 =^ 48%), although no statistically significant differences were observed between UAWD and placebo (MD = 9.8, 95% CI: [-2.55, 22.15], P = 0.12) (**Figure 6**).

### Effectiveness of treatment

**Figure 7:**
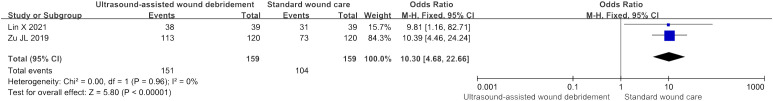
Forest plots of effectiveness of treatment.

Two studies (18, 19) had outcome measures associated with effectiveness of treatment, and the meta-analysis showed that UAWD had better effect than SWC (OR = 10.3, 95% CI: [4.68, 22.66], P < 0.00001, I^2 =^ 0%) (**Figure 7**).

### Wound blood perfusion

**Figure 8:**
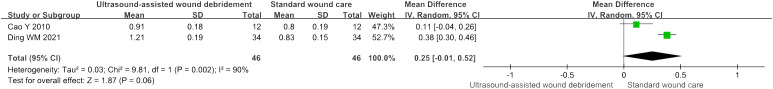
Forest plots of wound blood perfusion.

There were two studies (15, 16) that included wound blood perfusion as an observational indicator. Based on the results of meta-analysis, there were no statistically significant differences that were observed between UAWD and SWC (MD = 0.25, 95% CI: [-0.01, 0.52], P = 0.06, I^2 =^ 90%) (**Figure 8**).

### Transcutaneous oxygen partial pressure

**Figure 9:**
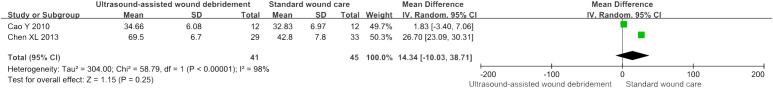
Forest plots of transcutaneous oxygen partial pressure.

Two studies (16, 17) investigated the effects of transcutaneous oxygen partial pressure by comparing UAWD with SWC, meta-analysis results showed no significant difference in two therapies (MD = 14.34, 95% CI: [-10.03, 38.71], P = 0.25, I^2 =^ 98%) (**Figure 9**).

### Adverse events

One study (12) assessed the safety of UAWD and placebo, in which 160 patients were reported to have adverse events such as cellulitis, development of additional wounds on the index foot, pain, wound drainage, and erythema. But these adverse events were proved to be unrelated to the two treatment measures. Adverse events were not mentioned in the other studies.

### Publication bias

Begg’s test and Egger’ s test were applied to evaluate the publication bias of each outcomes in this study. As displayed in the **Supplementary Files**, the P values calculated by using Begg’s test were more than 0.05, which illustrated that publication bias was inexistent. The detailed data is described in **Supplementary Material 3**.

## Discussion

The pathology of DFU is extraordinarily complex, due to the persistent hyperglycemic state and the associated complications that arise from it, including skin and soft tissue barrier disruption and infection, high oxidative stress, neuropathy, microangiopathy, and chronic inflammatory reaction (20), making it one of the most serious and expensive complications of diabetes to treat (21). Atherosclerosis is the pathological basis of many complications of diabetes, such as DFU and diabetes with coronary atherosclerotic heart disease. Many serum markers can predict the occurrence of these diseases to some extent (22). For example, high levels of serum homocysteine will damage blood vessels and significantly increase the risk of peripheral vascular disease, coronary heart disease and cerebrovascular disease in diabetes patients (23). The standard treatment for DFU is dressing therapy, plus offloading managing wound infection and sharp wound debridement would occurs if there is wound bed degeneration and or overlying/marginal tissue necrosis/biofilm. Wound debridement is a basic treatment for DFU, which includes surgical sharp debridement, enzymatic debridement, biological debridement, and autolysis debridement (24). Moreover, local wound decompression, diabetes related treatment and education (25), improving peripheral vascular circulation and regulating metabolism are also important treatment methods (26). Each of these methods has advantages and disadvantages. Surgical sharp debridement is commonly used, but it is not suitable for patients with poor vascular status, and most patients have multiple serious complications, and the wound surface has characteristics such as a wide range of diseased tissue, deep location, and scattered necrotic tissue. Traditional surgical debridement mainly relies on surgical knives or tissue scissors, resulting in significant damage and incomplete debridement, and sometimes can lead to exposure of the wound bones, joints and ligaments, which may cause certain damage to the wound bed (27). Some researchers believe that about 50% of patients with diabetes or DFU have peripheral artery disease, and it is estimated that 65% of DFUs have ischemic symptoms. Therefore, UAWD can be an effective alternative when sharp debridement is contraindicated, such as patients with poor vascular status (28). The principle of UAWD is mainly to use an ultrasonic debridement device for microcomputer automatic control, automatic frequency tracking, and output ultrasound. After pressurizing a sterile 0.9% sodium chloride solution to a certain pressure, a micro jet is generated through the handle nozzle, thereby generating a “cavitation” effect. By utilizing the pressure difference of toughness and elasticity of different tissues in the human body, necrotic tissue is accurately identified and removed without causing thermal damage to surrounding tissues, maximizing the protection of healthy tissue and structures; At the same time, it is believed to improve local blood flow perfusion and oxygen partial pressure, stimulate the release of growth factors and inflammatory mediators, promote the growth of fresh granulation tissue, and accelerate wound healing. UAWD can effectively remove necrotic tissue, biofilm, reduce wound bacterial count, improve granulation tissue survival rate and chronic wound healing rate. Its main mechanism of action is to stimulate the relevant signal transduction pathways of wound healing by the mechanical energy generated by ultrasound, promoting leukocyte adhesion, growth factor generation, fibroblast proliferation, and collagen production (29, 30). In addition, UAWD induces vibration and cavitation, resulting in sound flow and tiny bubbles along the boundary between sound waves and cell membranes, causing changes in cell function, enhancing protein synthesis, and increasing the permeability of cell membranes and vascular walls (31). Meanwhile, the increase in macrophage reactivity and the enhancement of fibrinolysis are also ultrasound induced cellular effects. Ultrasound energy is believed to decrease edema, inhibit bacterial colonization, reduce bacterial burden, and prevent biofilm formation (10). Therefore, in recent years, UAWD has been widely used in the clinical treatment of DFU and has achieved good therapeutic effects.

The meta-analysis results of this study showed that the subgroup analysis of wound healing rate showed that the UAWD group had a higher wound healing rate than the SWC group, and had a higher wound healing rate than the placebo group. The meta-analysis results of wound healing time showed that patients in the UAWD group (50.34 ± 13.61 days) had shorter wound healing time than those in the SWC group (57.64 ± 21.34 days), but there was significant heterogeneity in the data comparison between the two groups. Based on the information extracted from each study, sensitivity analysis did not find any significant sources of heterogeneity. In terms of treatment effectiveness, the UAWD group was significantly higher than the SWC group. As for wound area reduction rate, UAWD is significantly higher than SWC, but there is no statistically significant difference between UAWD and placebo. Similarly, in terms of wound blood flow perfusion and transcutaneous oxygen partial pressure, although the intra group comparisons of each study showed that UAWD was more effective than SWC, there was no significant statistical difference in the meta-analysis data between the UAWD group and SWC group.

This study contains the following limitations: firstly, we only included literature in Chinese and English, which may lead to certain regional limitations in the research results. Secondly, some of the included studies did not specify the Wagner level, duration of illness, and frequency values set for the use of ultrasound debridement machines in patients with DFU, which may lead to heterogeneity in research results due to differences in the severity of the patient’s condition and intervention measures. Thirdly, some studies have not reported the implementation of blinding, which may lead to a risk of bias. Fourthly, there is significant heterogeneity in wound healing time, and based on the information provided by the included studies, we do not currently know the source of heterogeneity. Fifthly, there are only three studies comparing UAWD with placebo, resulting in insufficient support for the reliability of research results.

## Conclusion

Based on the above results, it can be concluded that UAWD can significantly improve wound healing rate, shorten wound healing time, accelerate wound area reduction rate, and improve clinical treatment effectiveness without obvious adverse reactions. Although there is no significant difference in transcutaneous oxygen pressure and wound blood flow perfusion between UAWD and SWC. We look forward to more double blinded, placebo-controlled, high-quality studies in the future, To enable researchers to obtain more complete and accurate analytical data, in order to improve the scientific and credibility of the evidence.

## Data availability statement

The original contributions presented in the study are included in the article/**Supplementary Material**. Further inquiries can be directed to the corresponding author.

## Author contributions

EL: Writing – review & editing, Writing – original draft, Methodology, Investigation. XH: Writing – review & editing, Visualization, Software, Data curation. WZ: Writing – review & editing, Data curation. WX: Writing – review & editing, Validation, Methodology. YS: Writing – review & editing, Validation, Methodology. YL: Writing – review & editing, Data curation. ZZ: Writing – review & editing, Data curation. PZ: Writing – review & editing, Data curation. YH: Writing – review & editing, Software, Methodology. HQ: Writing – review & editing, Writing – original draft, Supervision, Resources, Project administration, Funding acquisition.
