# Supplementary material for: Efficacy and safety of ultrasound-assisted wound debridement in the treatment of diabetic foot ulcers: a systematic review and meta-analysis of 11 randomized controlled trials
Source: Front Endocrinol (Lausanne). 2024 May 1;15:1393251. doi: 10.3389/fendo.2024.1393251 (PMC11094243; doi:10.3389/fendo.2024.1393251)
Supplement: Supplementary file 1 [file DataSheet_1.docx]

Pubmed

("Diabetic Foot"[MeSH Terms] OR ("Diabetic Foot"[MeSH Terms] OR ("diabetic"[All Fields] AND "foot"[All Fields]) OR "Diabetic Foot"[All Fields] OR ("foot"[All Fields] AND "diabetic"[All Fields]) OR "foot diabetic"[All Fields] OR ("Diabetic Foot"[MeSH Terms] OR ("diabetic"[All Fields] AND "foot"[All Fields]) OR "Diabetic Foot"[All Fields] OR ("diabetic"[All Fields] AND "feet"[All Fields]) OR "diabetic feet"[All Fields]) OR ("Diabetic Foot"[MeSH Terms] OR ("diabetic"[All Fields] AND "foot"[All Fields]) OR "Diabetic Foot"[All Fields] OR ("feet"[All Fields] AND "diabetic"[All Fields]) OR "feet diabetic"[All Fields]) OR ("Diabetic Foot"[MeSH Terms] OR ("diabetic"[All Fields] AND "foot"[All Fields]) OR "Diabetic Foot"[All Fields] OR ("foot"[All Fields] AND "ulcer"[All Fields] AND "diabetic"[All Fields]) OR "foot ulcer diabetic"[All Fields]))) AND (("Ultrasound-Assisted"[All Fields] AND ("injuries"[MeSH Subheading] OR "injuries"[All Fields] OR "wounds"[All Fields] OR "wounds and injuries"[MeSH Terms] OR ("wounds"[All Fields] AND "injuries"[All Fields]) OR "wounds and injuries"[All Fields] OR "wound s"[All Fields] OR "wounded"[All Fields] OR "wounding"[All Fields] OR "woundings"[All Fields] OR "wound"[All Fields]) AND ("debride"[All Fields] OR "debrided"[All Fields] OR "debridement"[MeSH Terms] OR "debridement"[All Fields] OR "debridements"[All Fields] OR "debrides"[All Fields] OR "debriding"[All Fields] OR "debridment"[All Fields] OR "debridments"[All Fields])) OR (("ultrasonically"[All Fields] OR "ultrasonicated"[All Fields] OR "ultrasonication"[All Fields] OR "ultrasonicator"[All Fields] OR "ultrasonics"[MeSH Terms] OR "ultrasonics"[All Fields] OR "ultrasonic"[All Fields]) AND ("debride"[All Fields] OR "debrided"[All Fields] OR "debridement"[MeSH Terms] OR "debridement"[All Fields] OR "debridements"[All Fields] OR "debrides"[All Fields] OR "debriding"[All Fields] OR "debridment"[All Fields] OR "debridments"[All Fields]))) 123

| No. | Query | Results |
| --- | --- | --- |
| #1 | "Diabetic Foot"[Mesh] | 11709 |
| #2 | (((Foot, Diabetic) OR (Diabetic Feet)) OR (Feet, Diabetic)) OR (Foot Ulcer, Diabetic) | 20762 |
| #3 | #1 OR #2 | 20762 |
| #4 | ((Ultrasound-Assisted Wound Debridement) OR (ultrasonic debridement)) OR (ultrasound debridement) | 5912 |
| #5 | #3 AND #4 | 123 |

Embase

| No. | Query | Results |
| --- | --- | --- |
| #1 | 'diabetic foot'/exp OR 'diabetic foot' | 25429 |
| #2 | 'foot, diabetic':ab,ti OR 'diabetic feet':ab,ti OR 'feet, diabetic':ab,ti OR 'foot ulcer, diabetic':ab,ti | 426 |
| #3 | #1 OR #2 | 25493 |
| #4 | 'Ultrasound-Assisted Wound Debridement':ab,ti OR 'ultrasonic debridement':ab,ti OR 'ultrasound debridement':ab,ti | 211 |
| #5 | #3 AND #4 | 23 |

Web of science

| No. | Query | Results |
| --- | --- | --- |
| #1 | ((((TS=(Diabetic Foot)) OR TS=(Foot, Diabetic)) OR TS=(Diabetic Feet)) OR TS=(Feet, Diabetic)) OR TS=(Foot Ulcer, Diabetic) and Preprint Citation Index (Exclude – Database) | 31916 |
| #2 | ((TS=(Ultrasound-Assisted Wound Debridement)) OR TS=(ultrasonic debridement)) OR TS=(ultrasound debridement) and Preprint Citation Index (Exclude – Database) | 1824 |
| #3 | #1 AND #2 | 70 |

Cochrane library

| No. | Query | Results |
| --- | --- | --- |
| #1 | MeSH descriptor: [Diabetic Foot] explode all trees | 25429 |
| #2 | (Foot, Diabetic):ti,ab,kw OR (Diabetic Feet):ti,ab,kw OR (Feet,Diabetic):ti,ab,kw OR (Foot Ulcer, Diabetic):ti,ab,kw | 426 |
| #3 | #1 OR #2 | 25493 |
| #4 | (Ultrasound-Assisted Wound Debridement):ti,ab,kw OR (ultrasonic debridement):ti,ab,kw OR (ultrasound debridement):ti,ab,kw | 211 |
| #5 | #3 AND #4 | 28 |

CBM

| No. | Query | Results |
| --- | --- | --- |
| #1 | "糖尿病足"[不加权:扩展] | 27571 |
| #2 | "糖尿病足溃疡"[常用字段:智能] OR "糖尿病下肢溃疡"[常用字段:智能] OR "糖尿病性溃疡"[常用字段:智能] OR "糖尿病创面"[常用字段:智能] | 3540 |
| #3 | ("糖尿病足溃疡"[常用字段:智能] OR "糖尿病下肢溃疡"[常用字段:智能] OR "糖尿病性溃疡"[常用字段:智能] OR "糖尿病创面"[常用字段:智能]) AND ("糖尿病足"[不加权:扩展]) | 2903 |
| #4 | "超声清创"[常用字段:智能] OR "超声辅助清创"[常用字段:智能] | 130 |
| #5 | ("超声清创"[常用字段:智能] OR "超声辅助清创"[常用字段:智能]) AND (("糖尿病足溃疡"[常用字段:智能] OR "糖尿病下肢溃疡"[常用字段:智能] OR "糖尿病性溃疡"[常用字段:智能] OR "糖尿病创面"[常用字段:智能]) AND ("糖尿病足"[不加权:扩展])) | 69 |

CNKI

( SU %= '糖尿病足' OR TKA = '糖尿病足溃疡' OR TKA = '糖尿病下肢溃疡' OR TKA = '糖尿病性溃疡' OR TKA = '糖尿病创面') AND ( TKA = '超声清创' OR SU %= '超声辅助清创') 69

Wangfang Database

(主题:(糖尿病足) or 主题:(糖尿病足溃疡) or 主题:(糖尿病下肢溃疡) or 主题:(糖尿病性溃疡) or 主题:(糖尿病创面) ) and (主题:(超声清创) or 主题:(超声辅助清创)) 152

CQVIP Database

( R=糖尿病足 OR R=糖尿病足溃疡 OR R=糖尿病下肢溃疡 OR R=糖尿病性溃疡 OR R=糖尿病创面) AND (R=超声清创 OR R=糖尿病性溃疡) 40
