# Supplementary material for: Efficacy and safety of ultrasound-assisted wound debridement in the treatment of diabetic foot ulcers: a systematic review and meta-analysis of 11 randomized controlled trials
Source: Front Endocrinol (Lausanne). 2024 May 1;15:1393251. doi: 10.3389/fendo.2024.1393251 (PMC11094243; doi:10.3389/fendo.2024.1393251)

Sensitivity analysis of wound healing time

| Study omitted | Estimate | [95% Conf. Interval] |
| --- | --- | --- |
| Chen XL 2013 | -10.750608 | -24.873068 3.3718505 |
| Ding WM 2021 | -12.359322 | -26.013077 1.2944335 |
| Lázaro-Martínez JL 2020 | -10.114909 | -22.275976 2.046159 |
| Lin X 2021 | -12.462423 | -26.13348 1.2086344 |
| Michailidis, L 2018 | -17.519854 | -29.572418 -5.4672875 |
| Zu JL 2019 | -10.466135 | -14.643609 -6.288661 |
| Combined | -11.940264 | -23.653101 -.22742714 |


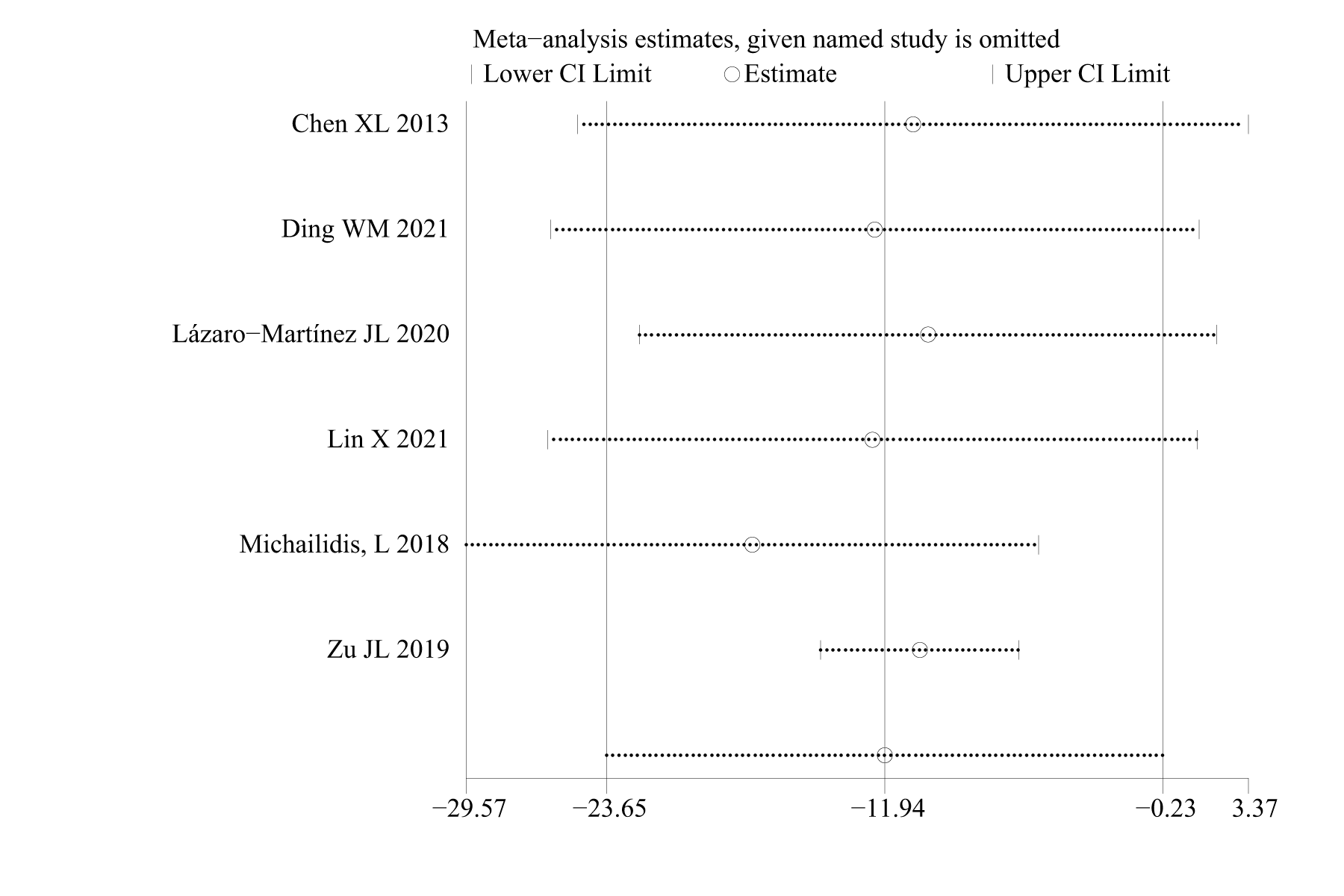

Supplement: Supplementary file 2 [file DataSheet_2.docx]
