# Supplementary material for: Efficacy and safety of ultrasound-assisted wound debridement in the treatment of diabetic foot ulcers: a systematic review and meta-analysis of 11 randomized controlled trials
Source: Front Endocrinol (Lausanne). 2024 May 1;15:1393251. doi: 10.3389/fendo.2024.1393251 (PMC11094243; doi:10.3389/fendo.2024.1393251)

**Publication bias**

| **Outcomes** | **Number of studies** | **Begg's test** | **Egger's test** |
| --- | --- | --- | --- |
| Healing rate | 8 | 0.711 | 0.646 |
| Wound healing time | 6 | 0.452 | 0.41 |
| Percentage reduction in wound size | 4 | 0.734 | 0.414 |
| Effectiveness of treatment | 2 | 1 | Not applicable |
| Wound blood perfusion | 2 | 1 | Not applicable |
| Transcutaneous oxygen partial pressure | 2 | 1 | Not applicable |

1. Healing rate


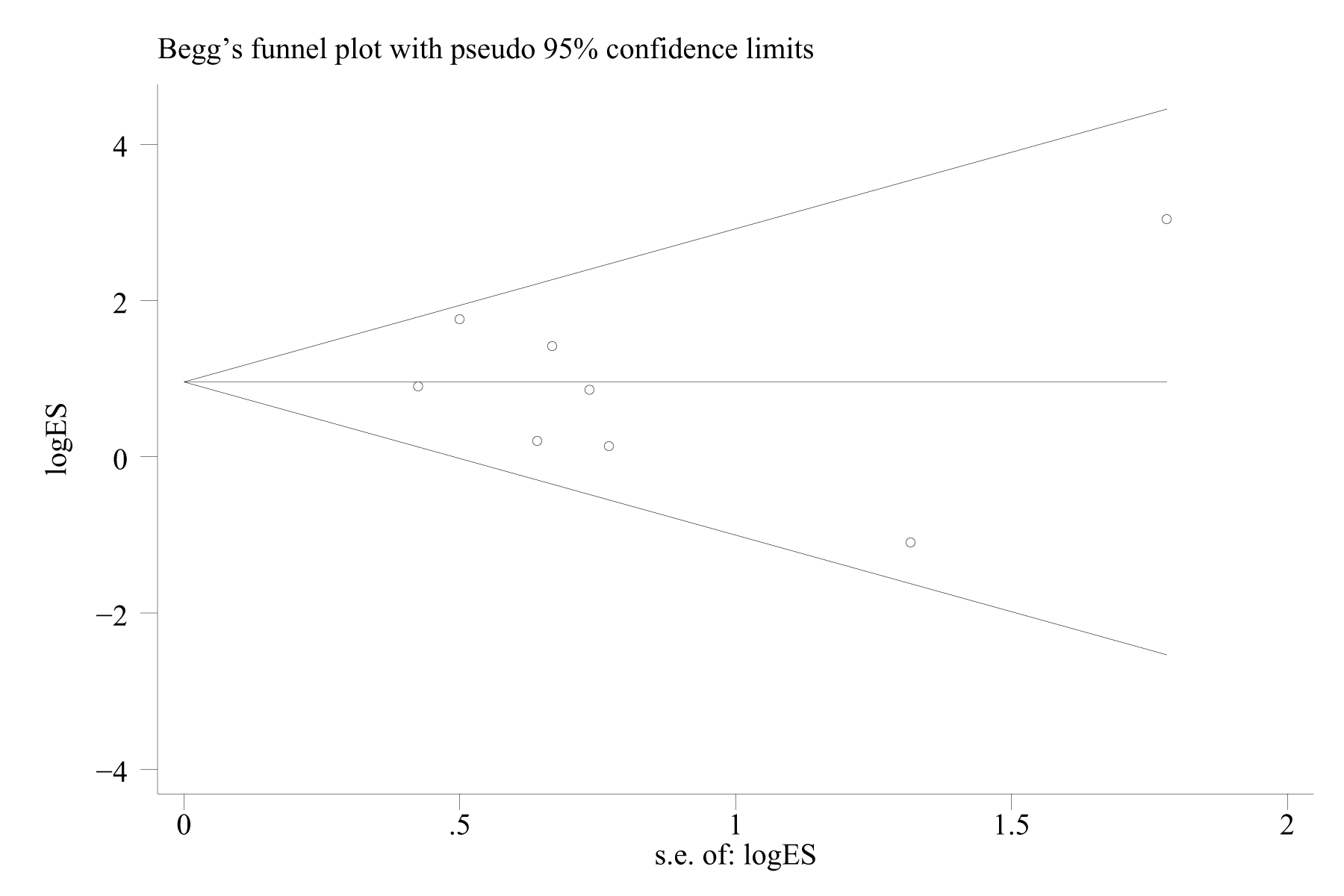


1. Wound healing time


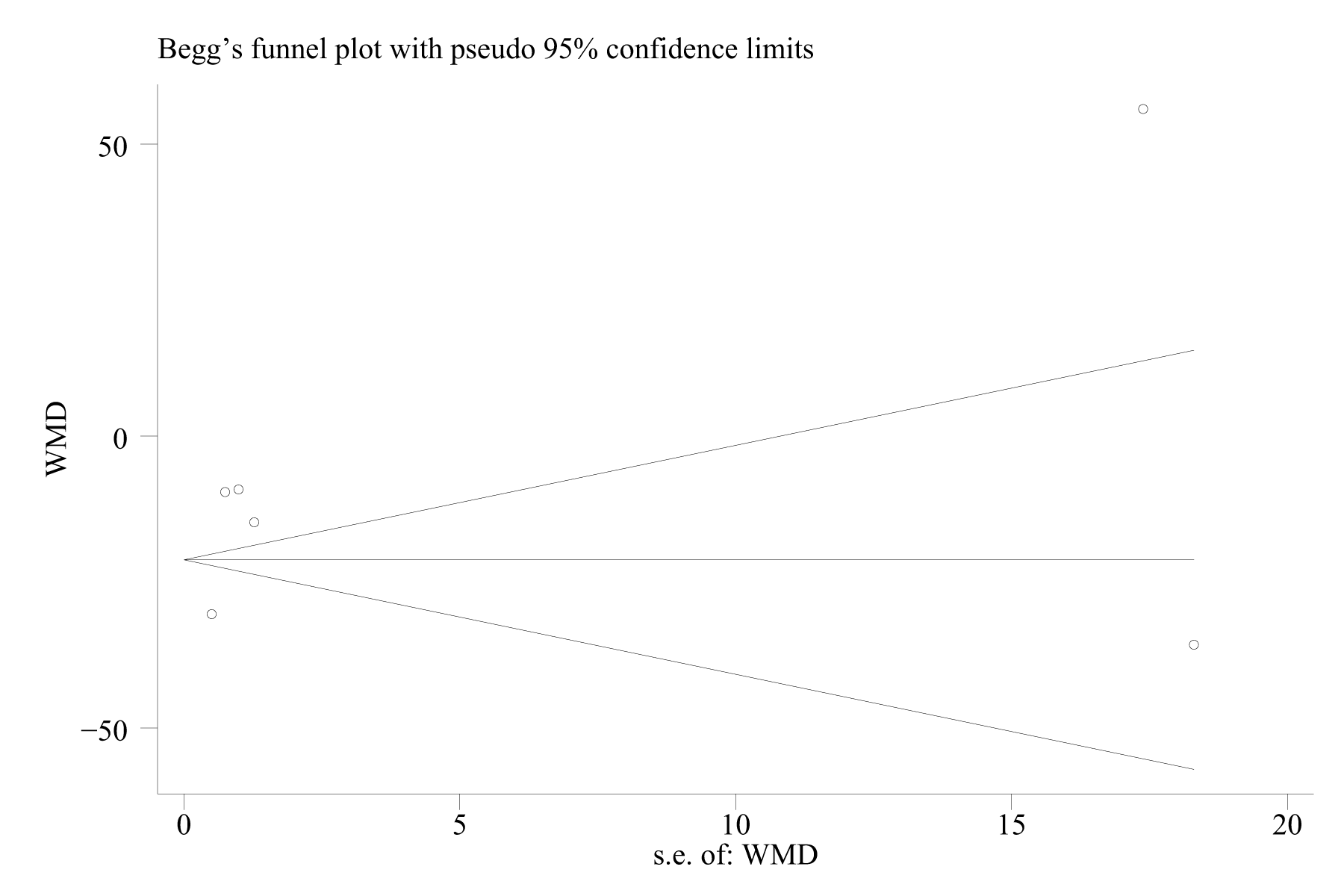


3.Percentage reduction in wound size


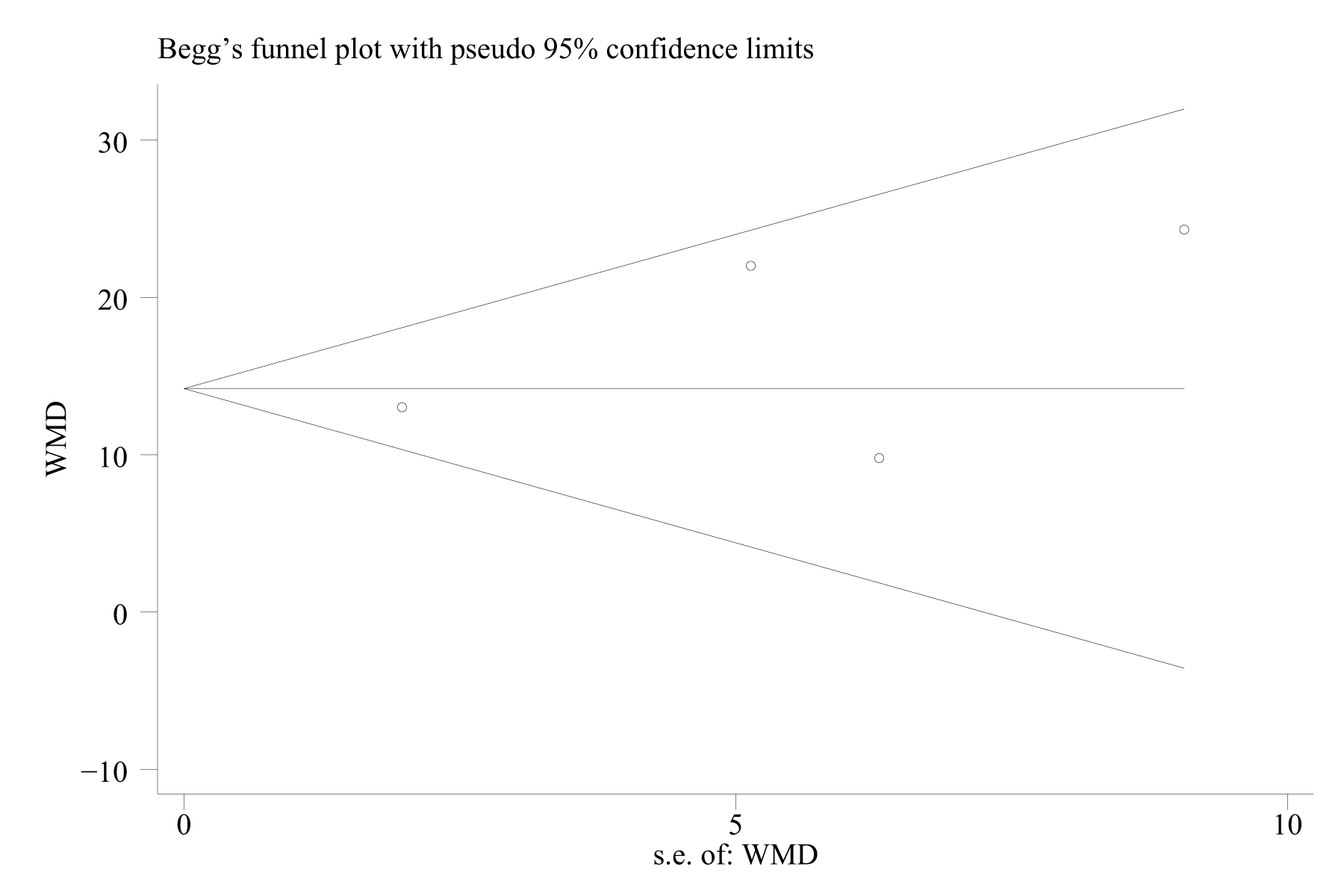


4.Effectiveness of treatment


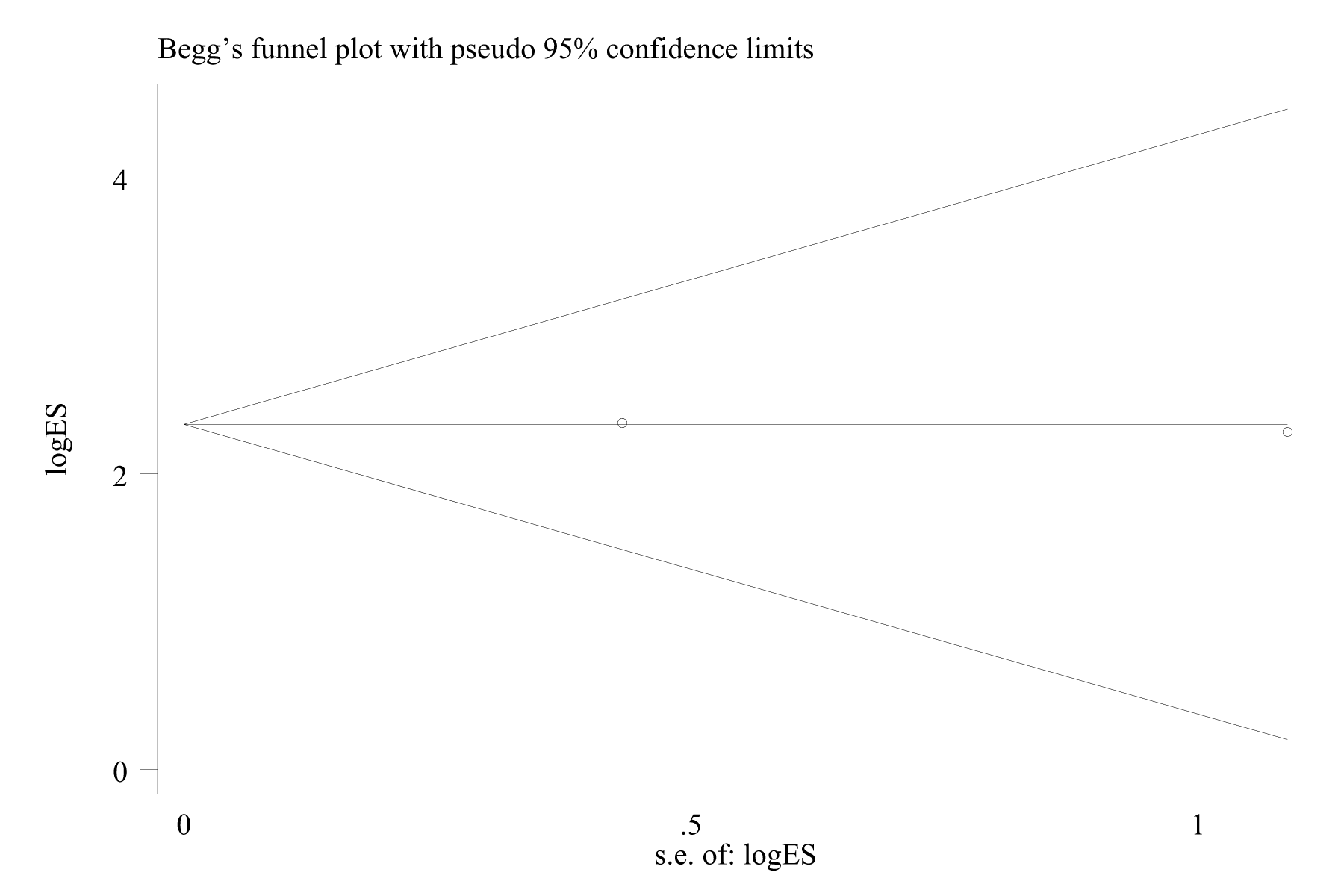


5.Wound blood perfusion


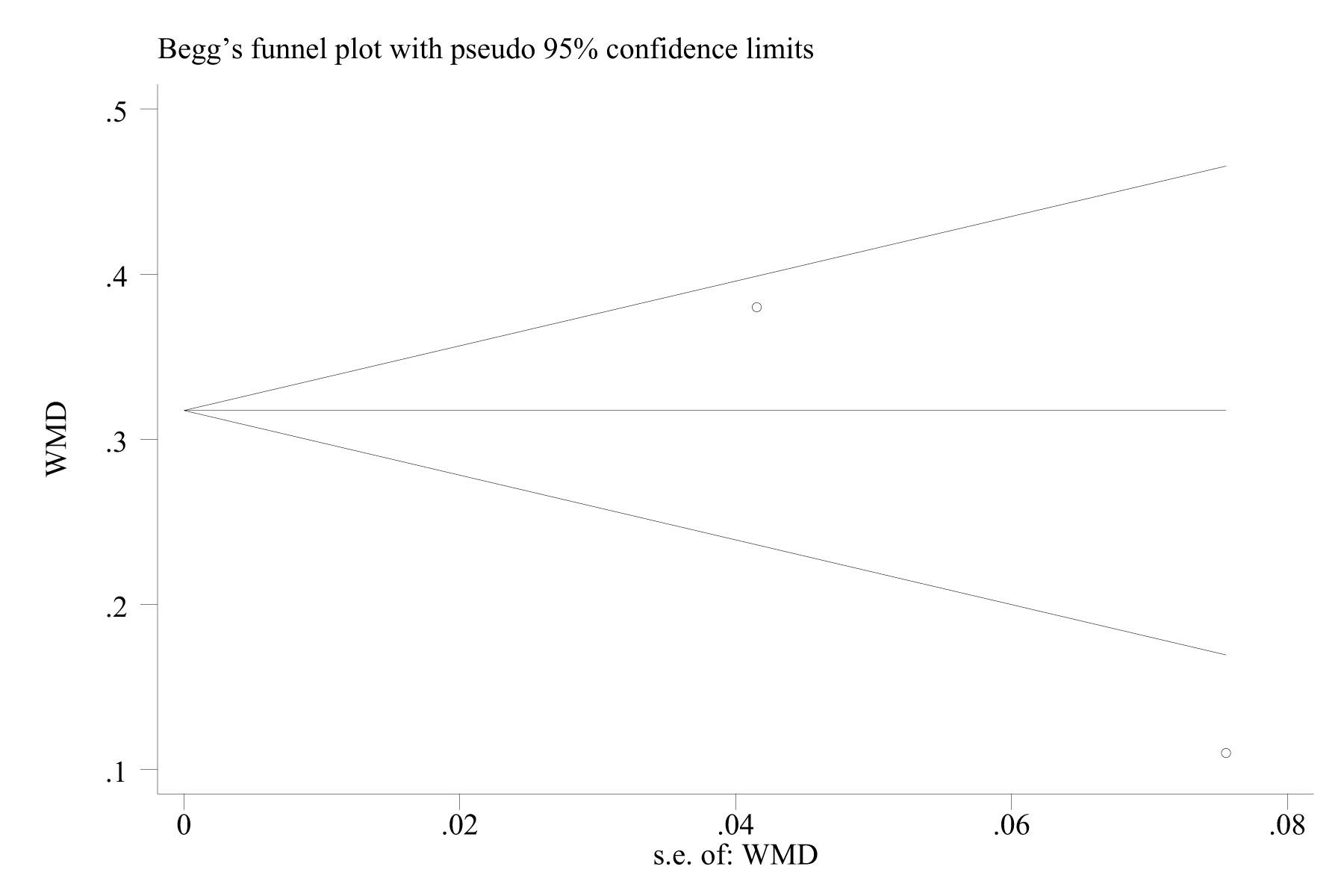


1. Transcutaneous oxygen partial pressure


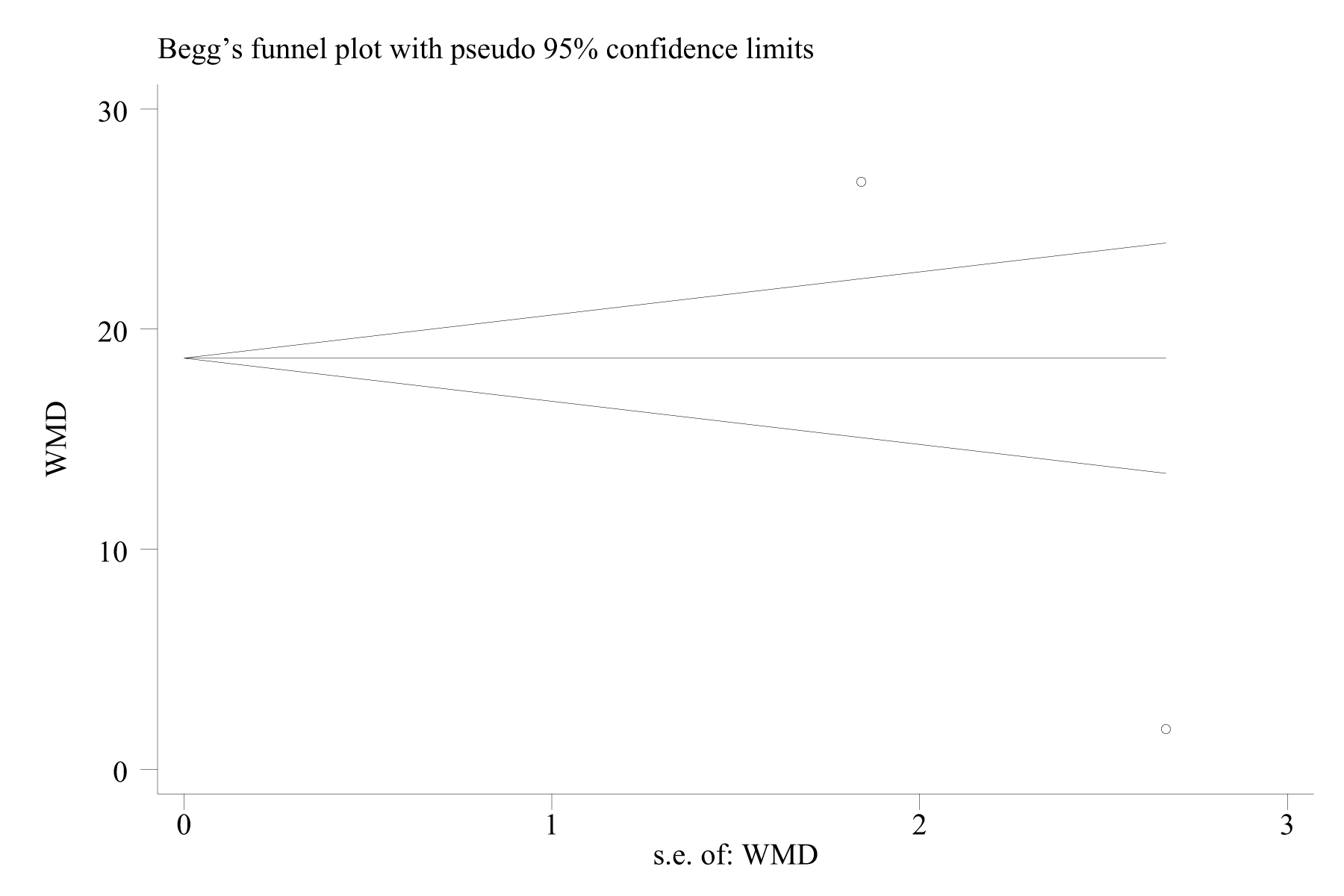

Supplement: Supplementary file 3 [file DataSheet_3.docx]
